# Adénocarcinome colique et carcinome à cellules rénales papillaire de type 1 synchrones: une association exceptionnelle

**DOI:** 10.11604/pamj.2017.27.28.12251

**Published:** 2017-05-10

**Authors:** Asma Sassi, Dhouha Bacha, Ghofrane Talbi, Sana Ben Slama, Rahma Boughriba, Zeineb Mzoughi, Saadia Bouraoui, Ahlem Lahmar

**Affiliations:** 1 Service d’Anatomie et Cytologie Pathologiques, Hôpital Mongi Slim, Sidi Daoued, 2046, Tunis, Tunisie; 2 Service de Chirurgie Générale, Hôpital Mongi Slim, Sidi Daoued, 2046, Tunis, Tunisie

**Keywords:** Cancer colorectal, carcinome à cellules rénales, cancers synchrones, Colorectal cancer, renal cell carcinoma, synchronous cancers

## Abstract

La découverte de tumeurs primitives synchrones à un cancer colorectal a fait l’objet de plusieurs publications. Cette association peut survenir de manière sporadique ou rentrer dans le cadre de syndromes cliniques bien définis tel que le syndrome de Lynch. L’association synchrone d’un cancer colorectal (CCR) et d’un carcinome à cellules rénales est rare. Elle est encore plus rare lorsque le carcinome à cellules rénales est de sous-type papillaire avec seulement 2 cas rapportés dans la littérature. L’association CCR et rénaux ne semble pas être liée à une anomalie des protéines du système MMR (MisMatch Repair) et n’intègre à ce jour aucun syndrome clinique. Nous rapportons le cas d’une femme âgée de 69 ans qui a simultanément présenté un adénocarcinome colique et un carcinome papillaire du rein de type 1, de découverte fortuite au cours du bilan d’extension du CCR. Nous discuterons la pathogénie ainsi que le pronostic de cette entité rarement décrite.

## Abstract (en)

The detection of primary tumors synchronous with colorectal cancer has been the subject of multiple publications. This association can occur sporadically or fall under the framework of well defined clinical syndromes such as Lynch syndrome. Synchronous association of colorectal cancer (CRC) and renal cell carcinoma is rare. It is even more rare when renal cell carcinoma is of papillary origin, with only 2 cases reported in the literature. The association between CRC and renal cell carcinoma does not seem to be related to mismatch repair proteins (MMR) abnormality and does not include, up to now, any clinical syndrome. We report the case of a 69-year old woman with colorectal cancer associated with synchronous type 1 papillary renal cell carcinoma unexpectedly detected during screening for CRC. We here discuss the pathogenesis as well as the prognosis of this rarely described entity.

## Introduction

L’association de tumeurs malignes primitives chez un même patient est rare.Dans la plupart des cas, le risque pour qu’un patient traité pour un cancer en développe un deuxième serait égal à celui de la population générale [1]. Cependant, cette possibilité soulève la question d’une éventuelle carcinogenèse commune, impliquant à la fois des mécanismes héréditaires, immunitaires et environnementaux. La découverte synchrone d’un cancer colique et d’autres tumeurs primitives a fait l’objet de plusieurs publications, rentrant soit dans le cadre de syndromes cliniques bien définis comme le syndrome de Lynch ou survenant de manière sporadique. L’association synchrone d’un carcinome colique et d’un carcinome à cellules rénales est rare. Nous rapportons un nouveau cas où le carcinome rénal était de type papillaire.

## Patient et observation

**Figure 1:**
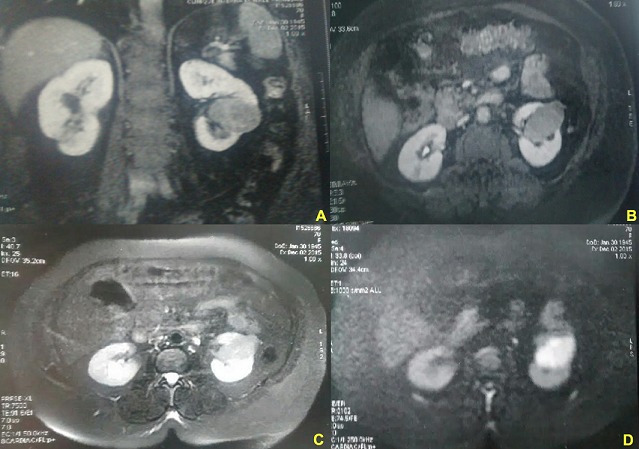
IRM rénale: tumeur médio-rénale gauche en hyposignal T1 (A), en signal intermédiaire T1 après injection de gadolinium (B), en hyposignal T2 (C) et en hypersignal sur la séquence de diffusion (D)

**Figure 2:**
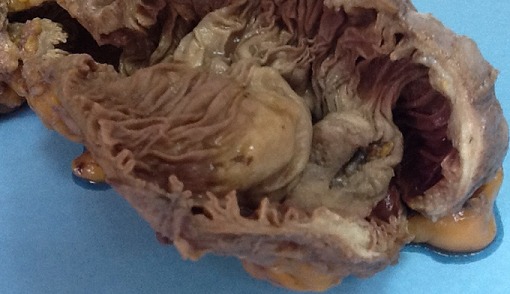
Pièce d’hémicolectomie droite fixée et ouverte: masse valvulaire, hémi-circonféretielle et ulcéro-bourgeonnante

Il s’agit d’une femme âgée de 69 ans, sans antécédents personnels ni familiaux de cancer, qui consultait pour des mélénas, évoluant depuis une semaine. L’examen clinique montrait un état général conservé et l’absence de masse abdominale palpable. Les aires ganglionnaires étaient libres. La fibroscopie œsogastroduodénale montrait une muqueuse gastrique érythémateuse et l’absence de saignement actif. La coloscopie notait la présence d’une formation ulcéro-bourgeonnante du caecum de 4cm de grand axe. L’examen histologique des biopsies de la masse caecale concluait à un adénocarcinome colique peu différencié et infiltrant. Dans le cadre du bilan d’extension, la tomodensitométrie thoraco-abdomino-pelvienne a révélé la présence d’une masse tissulaire médio-rénale gauche. Celle-ci mesurait 5 cm de grand axe et était arrondie, régulière, se réhaussant de manière hétérogène après l’injection de produit de contraste. Il n’a pas été observé par ailleurs d’autres anomalies notamment à l’étages thoraco-abdominal. Le diagnostic de métastase rénale du carcinome colique a été suspecté. L’imagerie par résonnance magnétique (IRM) rénale montrait que la masse était hypovasculaire, en hyposignal sur les séquences T1 et T2, en signal intermédiaire sur la séquence T1 après injection de gadolinium et en hypersignal sur la séquence de diffusion (Figure 1). Cet aspect était plutôt en faveur d’une tumeur rénale primitive. La patiente a été opérée par laparotomie. Une hémicolectomie droite carcinologique associée à une néphrectomie gauche élargie ont été pratiquées. Les suites opératoires étaient simples et la patiente était mise sortante à j5 post-opératoire. A l’examen macroscopique de la pièce colique, la tumeur était au niveau de la valvule de Bauhin. Elle était hémi-circonférentielle, d’aspect ulcéro-bourgeonnant et mesurait 3 cm de hauteur (Figure 2). Le curage n’avait ramené que 8 ganglions malgré la reprise des prélèvements après une fixation plus prolongée de la pièce.

**Figure 3:**
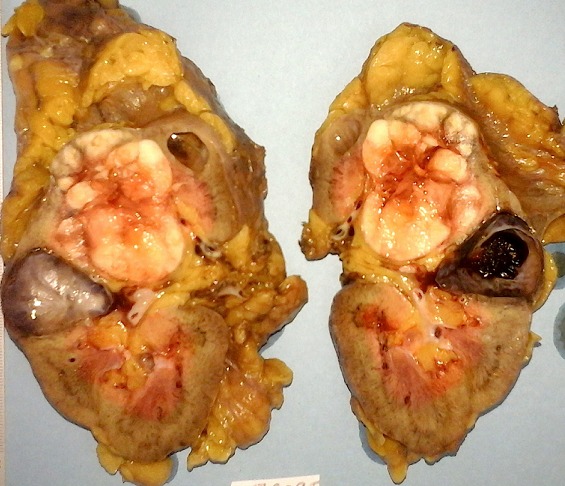
Pièce de néphrectomie gauche fixée et coupée selon son grand axe: tumeur blanc jaunâtre, polaire supérieure, de contours lobulés

**Figure 4:**
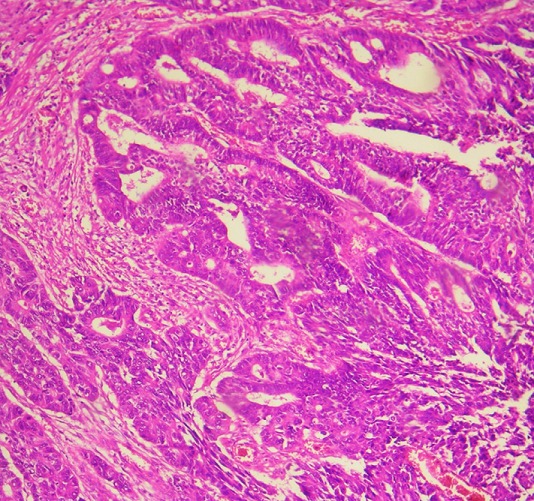
Adénocarcinome colique moyennement différencié: structures glandulaires de taille et de forme variées (hématoxylline éosine, grossissement x250)

Au niveau du rein, la tumeur était polaire supérieure et mesurait 5 x 4 x 4 cm. Elle était ferme, bien limitée, à contours lobulés, d’aspect homogène et blanc jaunâtre à la coupe (Figure 3). La tumeur arrivait au contact de la capsule rénale et de la graisse péri-hilaire mais sans signes d’infiltration de ces structures. A l’examen histologique, la tumeur de la valvule iléo-caecale correspondait à un adénocarcinome moyennement différencié, infiltrant la sous-séreuse, sans la dépasser (Figure 4). Il n’a pas été observé de signes d’invasion vasculaire ou nerveuse et les ganglions étaient réactionnels, sans signes de métastases. La tumeur était ainsi classée en pT3N0 (stade II) selon la classification pTNM dans sa dernière édition de 2009. Une étude immunohistochimique (IHC) a été effectuée à la demande des chirurgiens pour décider d’une éventuelle chimiothérapie adjuvante. Les anticorps testés étaient dirigés contre les protéines de réparation des mésappariements de l’ADN (ou protéines du système MMR pour mismatch repair) MLH1, MSH2, MSH6 et PMS2. Il n’a pas été observé de perte d’expression de ces protéines dans les cellules carcinomateuses et la tumeur était considérée de phénotype MSS (Micro-Satellite Stable).

**Figure 5:**
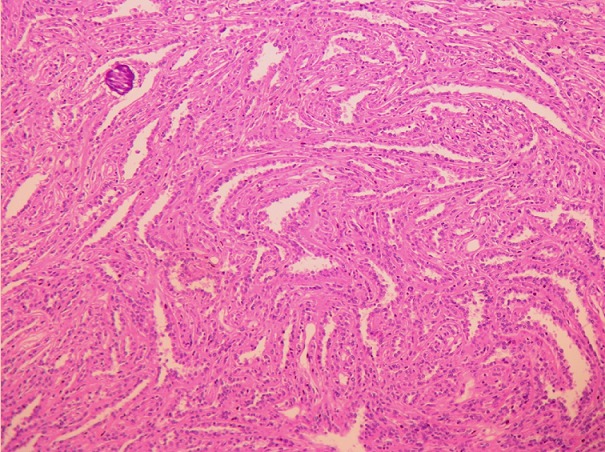
Carcinome rénal papillaire de type 1: structures papillaires centrées par un axe conjonctivo-vasculaire fin avec présence d’un corps psammomateux (hématoxylline éosine, grossissement x250)

Pour la tumeur rénale, elle correspondait à un carcinome papillaire de type 1, de grade nucléolaire 2 de l’ISUP. Il n’a pas été noté de signes d’invasion capsulaire ou vasculaire. La graisse hilaire et péri-rénale étaient indemnes. La tumeur était ainsi classée en pT1bNx selon la classification pTNM, éditée en 2010 (Figure 5). La patiente a reçu une chimiothérapie adjuvante à base de Folfox pendant 6 cures. L’évolution était favorable, sans signe de récidive ou de métastase, après 12 mois de suivi.

## Discussion

Les carcinomes colorectaux s’associent dans 5% des cas à des cancers extra-digestifs[2]. Selon Shiozawa et al, le siège de ces cancers diffère selon le sexe. En effet, chez l’homme, le cancer de l’estomac est le plus rapporté en association avec les CCR, suivi du cancer du poumon, de la prostate, de la région cervico-faciale, de l’œsophage et du rein [3]. Chez la femme, c’est le cancer du sein qui est le plus rapporté, suivi du cancer de l’utérus, de l’estomac et du poumon [3].

L’association des CCR avec ceux rénaux est extrêmement rare. Des séries autopsiques ayant étudié l’incidence des cancers associés au CCR avaient démontré que le cancer du rein était retrouvé dans seulement 0.046 à 0.1 % des cas [4, 5]. O’Boyle et Kemeny avaient retrouvé 6 cas de carcinome colique associé à un carcinome rénal parmi 1200 cas de CCR soit une incidence de 0.5% [6]. A ce jour, la plus grande étude, menée dans un centre expert d’oncologie sur une période de 40 ans, avait porté sur 24.642 patients suivis pour un CCR et 7366 patients suivis pour un carcinome rénal. Elle avait démontré, à l’instar des autres séries, la rareté de l’association: cancers colorectaux et rénaux. En effet, seulement 0.73% des CCR étaient associés à un carcinome rénal et 2.4% des carcinomes rénaux étaient associés à un CCR [7].

L’association carcinomes rénaux et autres cancers a également fait l’objet de plusieurs études épidémiologiques avec une incidence variant de 4.5 à 16.1% [8–12]. Beisland et al.avaient étudié le siège de ces cancers à travers 287 cas. Par ordre de fréquence, la prostate, la vessie, le poumon, le sein et le colon étaient les localisations les plus rapportées [12]. La plupart des carcinomes coliques et rénaux étaient métachrones [7, 12–17]. Dans ce cadre, le carcinome rénal était habituellement de découverte fortuite, lors du bilan d’extension radiologique, comme c’était le cas chez notre patiente, ou au cours du suivi post-thérapeutique [13–17].

L’étiopathogénie exacte de cette association est encore mal élucidée. Cullinane et al. avaient démontré à travers une série rétrospective de 7 patients porteurs de carcinomes synchrones rectaux et rénaux que les anomalies des protéines du système MMR ne seraient pas incriminées dans la genèse de cette association [18]. La recherche d’une instabilité micro-satellitaire était démontrée par phénotypage MSIà l’aide d’un panel de 10 marqueurs microsatellitaires. Seulement 5 blocs de paraffine pour l’adénocarcinome colique et 6 blocs de paraffine pour le carcinome rénal étaient disponibles [18]. Pour les carcinomes aussi bien rectaux que rénaux, le statut MSI était affirmé par la présence d’une instabilité d’au moins un marqueur. Le statut MSS était retenu en l’absence d’instabilité de tous les marqueurs. Les différents résultats sont résumés dans le Tableau 1. Quatre adénocarcinomes rectaux (80%) et cinq tumeurs rénales (83.33%) étaient de phénotype MSS. Un adénocarcinome rectal était de phénotype MSI. Les résultats étaient non concluants pour un carcinome rénal [18]. L’adénocarcinome rectal qui était de phénotype MSI était sporadique et ne remplissait pas les critères d’Amsterdam requis pour le diagnostic de syndrome de Lynch. De plus, le carcinome rénal associé était de phénotype MSS.

**Tableau 1:** Résultats de l’étude du statut MSI publiée par *Cullinane et al*. *[* *18* *].*

Patient n°	Siège de la tumeur	Type histologique	Stade TNM	Résulat MSI
1	Rectum	Adénocarcinome	T1N0M0	MSS
	Rein	CPR	T1N0M0	MSS
2	Rectum	Adénocarcinome	T3N0M1	MSS
	Rein	CCCR	T3N0M0	MSS
3	Rectum	Adénocarcinome	T2N0M0	MSI-H
	Rein	Oncocytome	N.A	MSS
4	Rectum	Adénocarcinome	T2N0M0	MSS
	Rein	CCCR	T3bN0M0	MSS
5	Rectum	Adénocarcinome	T1N0M0	MSS
	Rein	CCCR	T1N0M0	___^b^
6	Rectum	Adénocarcinome	T1N0M0	___
	Rein	CCCR	T2N0M0	N.C
7	Rectum	Adénocarcinome	T3N2M1	___
	Rein	CCCR	T1N0M0	MSS

CPR: Carcinome papillaire du rein; CCCR: carcinome à cellules claires du rein; N.A: non applicable; N.C: non concluant.

b blocs de paraffine non disponibles

Notre cas était de phénotype MSS pour l’adénocarcinome colique, ce qui est superposable aux données de la littérature. Néanmoins et faute de protocole, nous n’avons pas pu pratiquer une étude immunohistochimique pour préciser le statut MSI du carcinome papillaire du rein. Ces données suggèrent que les carcinomes rénaux associés aux CCR n’appartiennent pas au spectre lésionnel définissant le syndrome de Lynch, contrairement aux carcinomes des voies urinaires.

Les patients présentant l’association CCR et rénal auraient un risque accru de développer d’autres tumeurs malignes par rapport à la population générale, suggérant une possible prédisposition génétique [7]. Steinhagen et al avaient démontré, à travers une série portant sur 101 patients porteurs de CCR et rénaux, que 32%, 7% et 3% parmi eux présentaient respectivement une, deux et trois autres localisations carcinomateuses [7]. La troisième tumeur maligne la plus retrouvée était le cancer de la prostate pour l’homme avec une incidence de 15.5% et le cancer du sein pour la femme avec une incidence de 21%. Ces cancers étaient découverts à un stade précoce grâce à la surveillance rapprochée de ce groupe de patients [7].

Parmi les études ayant porté sur les associations CCR et cancers du rein, neuf seulement se sont intéressées aux types histologiques propres à cette entité.Ces séries avaient porté sur un total de 15 patients [13–16, 18–22] (Tableau 2). Dans ces séries, les CCR étaient tous des adénocarcinomes, de siège souvent rectal (9 cas) et colique droit (3 cas). Pour les 3 autres cas, les tumeurs étaient de siège colique gauche dans 2 cas et colique transverse dans un cas [13–16, 18–22]. Ces tumeurs infiltraient la muqueuse dans 6 cas, la musculeuse dans 3 cas, la sous-séreuse dans 4 cas et la séreuse dans 2 cas. Les carcinomes rénaux étaient principalement des carcinomes à cellules claires [13–15, 18–21]. Le carcinome papillaire du rein, comme celui observé chez notre patiente, était rapporté dans seulement 2 cas, associés tous les 2 à des carcinomes rectaux et non coliques [16, 18]. Thompson et al. avaient constaté que les patients porteurs d’un carcinome papillaire du rein, en association avec des CCR, étaient les plus susceptibles de développer une troisième tumeur maligne [1].

**Tableau 2:** Principales particularités anatomo-cliniques des cas d’association synchrone entre les carcinoma colorectaux et ceux à cellules rénales, rapportés dans la littérature

Auteur	Age/Sexe	Tumeur colique/rectale			Tumeur rénale		
		Siège	Type histologique	Stade	Localisation	Type histologique	Stade
***Kozokic***[19]	81/H	Sigmoïde	Adénocarcinome	pT2N0Mx	Droite	Carcinome à cellules claires grade ISUP 2.	N.P
***Amoroso***[20]	75/F	Colon droit	Adénocarcinome peu différencié	pT4aN0Mx	Droite	Carcinome à cellules claires	pT3aNxMx
***Spajic***[21]	69/H	Rectum	Adénocarcinome	pT1NxMx	Bilatérale	Carcinome à cellules claires grade ISUP 2.	N.P
***Cullinane***[18]	N.P	Rectum	Adénocarcinome	pT1N0M0	N.P	Carcinome papillaire	pT1N0M0
		Rectum	Adénocarcinome	pT3N0M1		Carcinome à cellules claires	pT3N0M0
		Rectum	Adénocarcinome	pT2N0M0		Oncocytome	N.A
		Rectum	Adénocarcinome	pT2N0M0		Carcinome à cellules claires	pT3bN0M0
		Rectum	Adénocarcinome	pT1N0M0		Carcinome à cellules claires	pT1N0M0
		Rectum	Adénocarcinome	pT1N0M0		Carcinome à cellules claires	pT2N0M0
		Rectum	Adénocarcinome	pT3N2M1		Carcinome à cellules claires	pT1N0M0
**Takahashi**[15]	70/F	Colon droit	Adénocarcinome moyennement différencié	pT3N2aM0	Droite	Carcinome à cellules claires	pT1bN0M0
**Veenstra**[14]	70/F	Colon droit	Adénocarcinome moyennement différencié	pT3N1Mx	Gauche	Carcinome à cellules claires grade ISUP 4.	pT3aNxMx
**Piao** [16]	63/H	Rectum	Adénocarcinome	ypT1NxMx	Gauche	Carcinome papillaire de type 1	pT1aNxMx
**Dalli** [22]	60/H	Colon gauche	Adénocarcinome moyennement différencié	pT4N0Mx	Gauche	Oncocytome	N.A
**Dafashy**[13]	36/F HNPCC	Colon transverse	Adénocarcinome moyennement différencié	pT1N1M0	Gauche	Carcinome à cellules claires grade ISUP 4.	pT2aN1Mx
**Notre cas**	69/F	Caecum	Adénocarcinome moyennement différencié	pT3N0	Gauche	Carcinome papillaire de type 1	Pt1bNxMx

H: homme; F: femme ; N.P:non précisé *;* N.A: non applicable; HNPCC: Hereditarynonpolyposis colorectal cancer

Les carcinomes rénaux, lorsqu’ils sont de découverte fortuite, comme c’était le cas de notre patiente, seraient de meilleur pronostic que les formes symptomatiques avec un grade et un stade histologiques moins avancés et des taux de survie à 5 ans significativement plus élevés (85,3% vs 62.5%) [23].

## Conclusion

L’association synchrone d’un CCR et d’un carcinome à cellules rénales est rare. Elle est souvent de phénotype MSS et ne rentre pas dans le cadre d’un syndrome de Lynch. Le risque accru de survenue d’autres cancers, en dehors des 2 cités, est bien démontrée et justifie une surveillance rigoureuse de ces patients. Ceci incite à rechercher un cadre syndromique associant les CCR et ceux du rein. La découverte d’un éventuel substrat génétique de ce syndrome nécessitera de larges études multicentriques.

## Conflits d’intérêts

Les auteurs ne déclarent aucun conflit d’intérêt.
